# Surface Thermodynamics, Viscosity, Activation Energy of N-Methyldiethanolamine Aqueous Solutions Promoted by Tetramethylammonium Arginate

**DOI:** 10.3390/e22121337

**Published:** 2020-11-25

**Authors:** Xiangfeng Tian, Lemeng Wang, Pan Zhang, Dong Fu

**Affiliations:** 1 School of Environmental Science and Engineering, North China Electric Power University, Baoding 071003, China; tianxiangfeng@ncepu.edu.cn (X.T.); zhangpan01@ncepu.edu.cn (P.Z.); fudong@tsinghua.org.cn (D.F.); 2 Hebei Key Lab of Power Plant Flue Gas Multi-Pollutants Control, Department of Environmental Science and Engineering, North China Electric Power University, Baoding 071003, China

**Keywords:** surface thermodynamics, viscosity, MDEA, [N_1111_][Arg]

## Abstract

The surface tension and viscosity values of N-methyldiethanolamine (MDEA) aqueous solutions promoted by tetramethylammonium arginate ([N_1111_][Arg]) were measured and modeled. The experimental temperatures were 303.2 to 323.2 K. The mass fractions of MDEA (*w*_MDEA_) and [N_1111_][Arg] (*w*_[N1111][Arg]_) were 0.300 to 0.500 and 0.025 to 0.075, respectively. The measured surface tension and viscosity values were satisfactorily fitted to thermodynamic models. With the aid of experimentally viscosity data, the activation energy (*E*a) and H_2_S diffusion coefficient (*D*_H2S_) of MDEA-[N_1111_][Arg] aqueous solution were deduced. The surface entropy and surface enthalpy of the solutions were calculated using the fitted model of the surface tension. The quantitative relationship between the calculated values (surface tension, surface entropy, surface enthalpy, viscosity, activation energy, and H_2_S diffusion coefficient) and the operation conditions (mass fraction and temperature) was demonstrated.

## 1. Introduction

China is a large coke producer, and coke oven gas (COG) is the second most abundant coking product after coke in the coking industry, with generation of 300–360 m^3^ of COG as a by-product for every 1 t of coke produced [1]. The main components of COG are hydrogen and methane, which have high calorific values and application value. COG can be used as an urban or industrial fuel gas, in gas-fired power stations to generate electricity, or as a raw material to synthesize ammonia, methanol, and other chemicals [2]. However, COG contains impurities that must be removed before use, such as hydrogen sulfide (H_2_S). As an odorous, toxic, and corrosive gas, H_2_S can cause severe corrosion of equipment and transportation pipelines, and its combustion products, SO_2_, can also cause environmental problems, such as acid rain [3]. Therefore, COG must be desulfurized to improve the gas quality and protect the environment.

Chemical absorption using alkanolamines as absorbents is a mature desulfurization method [4,5]. The alkanolamine method has the distinguishing feature of large absorption capacity, high removal efficiency, and good stability and reliability. The most widely used alkanolamine is monoethanolamine (MEA), which can remove more than 98% of H_2_S from COG [6]. However, the MEA method has certain disadvantages. For example, because of its corrosiveness, the MEA content is generally not allowed to exceed 30% in a solution, its regeneration process is highly energy-consuming, and it is volatile and prone to degradation, which results in large consumption during operation [7]. N-methyldiethanolamine (MDEA), a tertiary alkanolamine, is significantly less corrosive than MEA, exhibits strong resistance to degradation and oxidation, has low reaction heat, and its concentration in the solution can be high, which reduces the energy consumption of the solvent regeneration process. Studies have shown that MDEA has excellent selectivity and efficiency for the removal of H_2_S [8,9].

In addition to containing a single alkanolamine, an absorbent solution often contains two or three alkanolamines to combine the advantages of each while avoiding their drawbacks. Many studies have shown that a mixed alkanolamine solution has better desulfurization performance than a single alkanolamine solution [9,10,11]. Our previous study indicated that adding small quantities of MEA to MDEA aqueous solutions can obviously improve the H_2_S absorption capacity and absorption rate [12].

Ionic liquids (ILs) are considered a green solvent with many excellent properties and are attracting increased attention in the field of acid gas absorption [13,14,15,16,17,18,19,20,21]. However, ILs are highly viscous and their current prices are relatively high, which hinders their use as a pure solvent in large-scale commercial applications. Therefore, it is desirable to use ILs jointly with alkanolamines. Amino acid ionic liquids (AAILs), which are synthesized from amino acids, have advantages over ILs. Furthermore, they can be synthesized from widely available raw materials and, thus, their costs are much lower. As a result, they are often used in H_2_S removal studies [22,23,24]. In our previous studies, we used MDEA aqueous solutions promoted by two ILs, namely tetramethylammonium glycinate ([N_1111_][Gly]) and tetramethylammonium arginate ([N_1111_][Arg]), to explore the performances of such mixed solutions for absorbing H_2_S. The results showed that the two AAILs were superior to MEA in promoting the absorption of H_2_S by MDEA solution. Moreover, of these two promoters, the MDEA aqueous solutions promoted by [N_1111_][Arg] exhibited the highest absorption capacity and absorption rate for low-concentration H_2_S. Therefore, [N_1111_][Arg]-promoted MDEA aqueous solutions show commercial potential [12,25].

Viscosity and surface tension are the two main physical parameters of a solution, and significantly affect the mass transfer, heat transfer, and gas–liquid flow process [26,27,28,29]. They play a vital role in process simulations and the development of desulfurization equipment. Zuiderweg [30] reported that the surface tension has a greater effect on mass transfer processes than other physical properties, such as the density, viscosity, and diffusion coefficient. The smaller the surface tension, the smaller the mean diameter of the bubbles, which increases the interfacial mass transfer area [31]. If the desulfurization equipment is a tray column, the surface tension impacts the bubble size by affecting the bubble stability, which has an effect on the mass transfer area. A high solution viscosity promotes bubble accumulation, which leads to a decrease in the mass transfer efficiency [32]. Therefore, the determination of these thermodynamic properties is vital for practical applications of a solution. To date, no studies have been conducted on the measurement of the viscosity and surface tension values of MDEA-[N_1111_][Arg] solutions, or to investigate the effects of the temperature and solution concentration on the viscosity and surface tension.

To fill this knowledge gap, the viscosity and surface tension values of MDEA-[N_1111_][Arg] solutions under different mass fractions and temperatures were measured and modeled in this study. Based on the experimental data and calculation, the surface entropy (*S^S^*), surface enthalpy (*H^S^*), viscosity activation energy (*E*a), and H_2_S diffusion coefficient (*D*_H2S_) were obtained, and the effects of the temperature and solute mass fraction on these results were analyzed. To this end, the mass fractions of MDEA (*w*_MDEA_) and [N_1111_][Arg] (*w*_[N1111][Arg]_) in the solutions were changed from 0.300 to 0.500 and from 0.025 to 0.075, respectively, and the solution temperature was changed from 303.2 to 323.2 K.

## 2. Experimental

### 2.1. Reagents

The reagents used in the experiments are shown in Table 1. Each component in the absorbent was accurately weighed using an analytical balance (FA1604A, uncertainty = ±0.1 mg) based on the required mass percentages, and the components were well mixed.

### 2.2. Instrumentation and Process

Surface tension was determined by the BZY-1 surface tension meter (which employs the Wilhemy plate method, uncertainty = ±0.1 mN·m^−1^). The viscosity was determined by the NDJ-5S digital viscometer (uncertainty = ±0.1 mPa·s). The operational procedures and the reliability of the instruments were documented in our previous studies and are not repeated here [33,34,35].

## 3. Results and Discussion

### 3.1. Surface Tension and Model

Table 2 presents the surface tension values of the aqueous MDEA-[N_1111_][Arg] solutions at different mass fractions and temperatures. In addition to obtaining data experimentally, it is also important to develop an accurate model to fit and predict the surface tension values. The surface tension of a mixed solution depends on the composition and temperature of the solution. The model used in a previous study was adopted here because of its simplicity and prediction accuracy [36]:

(1) $$\gamma^{aq}\;=\;0^{\;}+\;\gamma \prime$$

where *γ*^0^ and *γ*′ can be expressed as follows:

(2) $$\gamma^{0}=x_{1}\gamma_{1}+x_{2}\gamma_{2}+x_{3}\gamma_{3}$$

(3) $$\gamma \prime =x_{1}x_{2}G_{12}+x_{1}x_{3}G_{13}+x_{2}x_{3}G_{23}$$

where the subscripts 1, 2, and 3 in the formulas represent MDEA, [N_1111_][Arg], and water, respectively; *x_i_* represents the mole fraction of component *i*; and *γ_i_* represents the surface tension of pure component *i*, which is linear with temperature. *G_ij_* represents the mutual influence between components *i* and *j*. To adapt to the new solution system in this study, the calculation equation is obtained by modifying the equation used in the previous research [36]:

(4) $$G_{13}=\left(a_{13}+b_{13}w_{\mathrm{MDEA}}\right)T$$

(5) $$G_{23}=\left(a_{23}+b_{23}w_{\left[N1111\right]\left[\mathrm{Arg}\right]}\right)T$$

(6) $$G_{12}=\left(a_{12}+b_{12}\left[\left(w_{\mathrm{MDEA}}+w_{\left[N1111\right]\left[\mathrm{Arg}\right]}\right)/2\right]\right)T$$

By combining Equations (1)–(6), the surface tension can be formulated as:

(7) $$\begin{array}{cc} \gamma^{aq}=\gamma^{0}+\gamma^{\prime }= & x_{1}\gamma_{1}+x_{2}\gamma_{2}+x_{3}\gamma_{3}+x_{1}x_{2}G_{12}+x_{1}x_{3}G_{13}+x_{2}x_{3}G_{23}\\ & =x_{1}\left(a_{1}T+b_{1}\right)+x_{2}\left(a_{2}T+b_{2}\right)+x_{3}\left(a_{3}T+b_{3}\right)+x_{1}x_{2}(a_{12}\\ & +b_{12}\left[\left(w_{\mathrm{MDEA}}+w_{\left[N1111\right]\left[\mathrm{Arg}\right]}\right)/2\right])T+x_{1}x_{3}\left(a_{13}+b_{13}w_{\mathrm{MDEA}}\right)T\\ & +x_{2}x_{3}\left(a_{23}+b_{23}w_{\left[N1111\right]\left[\mathrm{Arg}\right]}\right)T \end{array}$$

where $\gamma_{i}=\left(a_{i}T+b_{i}\right)$ represents the surface tension of pure component *i*, which varies linearly with temperature.

For a ternary solution, six adjustable model parameters should be optimized using experimental data so that the established thermodynamic model can provide accurate predictions. In the process of optimizing parameters, the average relative deviation (ARD) can be defined as follows:

(8) $$\mathrm{ARD}=\frac{1}{n}\overset{n}{\underset{i=1}{\sum }}\left[1-\gamma^{cal}/\gamma^{exp}\right]\cdot 100\%$$

The superscripts *cal* and *exp* represent the experimental and calculated results, respectively, and *n* is the number of experimental data. The optimized parameters were determined to be the following: *a*_13_ = −1.31, *b*_13_ = 1.59, *a*_23_ = −1.73, *b*_23_ = −0.866, *a*_12_ = 8.72, *b*_12_ = 11.5, and ARD = 1.23%. The small ARD value indicates that the predicted results fit well with the experimental results.

**Figure 1:**
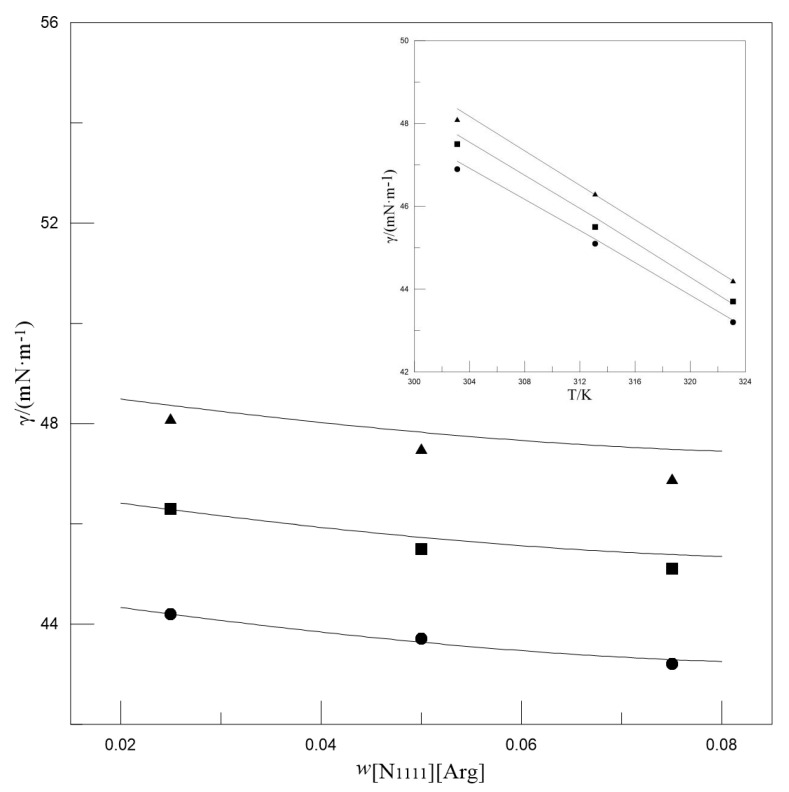
The effect of *w*_[N1111][Arg]_ (main plot) and temperature (inset plot) on the surface tensions of MDEA-[N_1111_][Arg] aqueous solutions. Main plot: *w*_MDEA_ = 0.500, ▲*T* = 303.2 K, ■*T* = 313.2 K, ●*T* = 323.2 K. Inset plot: *w*_MDEA_ = 0.500, ▲*w*_[N__1111][Arg]_ = 0.025, ■*w*_[N__1111][Arg]_ = 0.050, ●*w*_[N__1111][Arg]_ = 0.075. Symbols: Experimental values. Lines: Calculated values.

Figure 1 presents the changes in the surface tension of MDEA-[N_1111_][Arg] aqueous solutions with changes in the *w*_[N1111][Arg]_ and temperature. The surface tension gradually decreased as the temperature increased. This phenomenon may be because the molecular motion intensified as the temperature increased, which increased the kinetic energy and decreased the intermolecular cohesion, thereby reducing the surface tension [37]. In addition, as the *w*_[N1111][Arg]_ and *w*_MDEA_ increased, the surface tension showed a gradual decrease. This might be caused by the presence of alkyl groups in the solvent component, which makes them easier to distribute at the gas–liquid interface.

Moreover, the established model parameters can be used to calculate other surface thermodynamic properties of the solution, such as the surface entropy (*S^S^*) and surface enthalpy (*H^S^*) [35,38]:

(9) $$S^{s}=-{\left(\frac{\partial \gamma^{\mathrm{aq}}}{\partial T}\right)}_{x,\;P}$$

(10) $$H^{s}=\gamma -T{\left(\frac{\partial \gamma^{\mathrm{aq}}}{\partial T}\right)}_{x,\;P}$$

**Figure 2:**
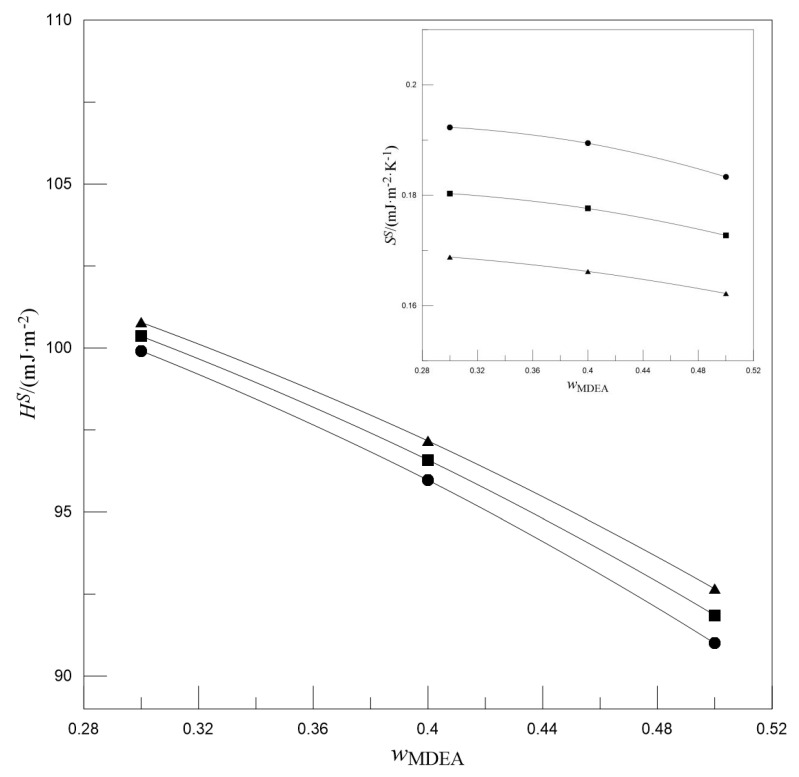
The effect of *w*_MDEA_ on the surface enthalpies (main plot) and surface entropies (inset plot). ▲*w*_[N__1111][Arg]_ = 0.025, ■*w*_[N__1111][Arg]_ = 0.050, ●*w*_[N__1111][Arg]_ = 0.075. Symbols: Experimental values. Lines: Trend lines.

**Figure 3:**
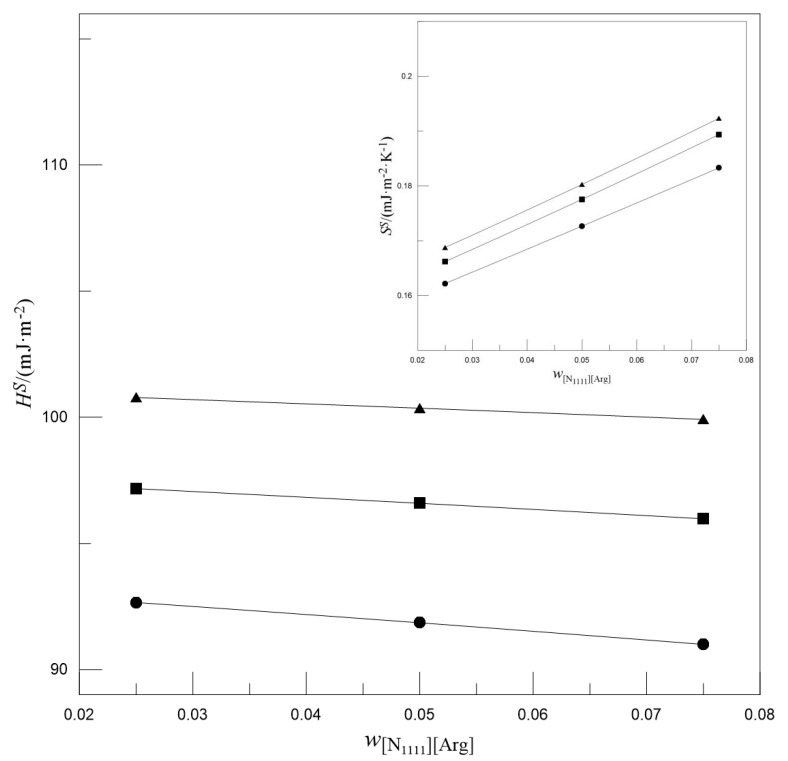
The effect of *w*_[N__1111][Arg]_ on the surface enthalpies (main plot) and surface entropies (inset plot). ▲*w*_MDEA_ = 0.3, ■*w*_MDEA_ = 0.4, ●*w*_MDEA_ = 0.5. Symbols: Experimental values. Lines: Trend lines.

Gliński et al. [39] and Maham et al. [40] fit the surface tension values of alkanolamine solutions to a linear function of temperature, *γ^aq^* = K_1_ + K_2_*T*. Therefore, for a given mass fraction of alkanolamine, the *S^S^* and *H^S^* values of the solution are −K_2_ and K_1_, respectively. However, in this study, the absorption solution is a ternary mixture and may not be appropriate using the above equation. Given that Equation (7) can be used to fit the surface tension, it can be used in conjunction with Equations (9) and (10) to further calculate the *S^S^* and *H^S^* values of the solution; the results are shown in Figure 2 and Figure 3.

Figure 2 shows the effect of *w*_MDEA_ on the *S^S^* and *H^S^*. For a given *w*_[N1111][Arg]_, the *S^S^* and *H^S^* values of the solutions both decrease with the increase in *w*_MDEA_. Figure 3 presents the effect of *w*_[N1111][Arg]_ on the *S^S^* and *H^S^*. For a given *w*_MDEA_, the *H^S^* values of the solutions decrease slightly with the increase in *w*_[N1111][Arg]_, whereas the *S^S^* values increase with the increase in *w*_[N1111][Arg]_. The decrease in *H^S^* indicates that less energy is required when the surface area increases. The increase in *S^S^* after adding [N_1111_][Arg] may be because its molecules are more likely to be distributed on the surface; after the molecules migrate to the surface, the intermolecular forces decrease, which leads to the decrease in the order degree of molecular arrangement.

### 3.2. Viscosity and the Model

The viscosity data of MDEA-[N_1111_][Arg] solutions were measured and results are shown in Table 3. Measuring all viscosity data is a highly expensive, time-consuming, and difficult process. An alternative means is to use an equation that can correlate the viscosities correctly. Numerous different equations have been proposed to correlate and predict the viscosity data of solutions [41,42,43,44]. Of these, the Weiland equation [44], which is a semi-empirical equation, can describe the dependence of solute composition and temperature on viscosity simultaneously. Thus, it is used for the correlation of viscosity data in this study [33,45]. For MDEA-[N_1111_][Arg] aqueous solutions, it can be expressed as:

(11) $$\eta_{mix}=\frac{w_{MDEA}}{w_{MDEA}+w_{\left[N_{1111}\right]\left[\mathrm{Arg}\right]}}\eta_{1}+\frac{w_{\left[N_{1111}\right]\left[\mathrm{Arg}\right]}}{w_{MDEA}+w_{\left[N_{1111}\right]\left[\mathrm{Arg}\right]}}\eta_{2}$$

where *η_mix_* represents the viscosity of MDEA-[N_1111_][Arg] solution, and *η*_1_ and *η*_2_ are expressed as:

(12) $$\eta_{i}=\eta_{water}\times \exp\left\{\frac{\left[\left(a_{i}w+b_{i}\right)T+\left(c_{i}w+d_{i}\right)\right]w}{T^{2}}\right\}$$

where *η_water_* represents the viscosity of pure water, *w* = *w*_MDEA_ + *w*_[N__1111][Arg]_, *a_i_*, *b_i_*, *c_i_*, *d_i_* are adjustable parameters. In this work, the parameters for MDEA were calculated by fitting to the viscosity data of MDEA aqueous solutions: (*a*_1_ = −0.1863, *b*_1_ = 0.3844, *c*_1_ = 879.8408, *d*_1_ = 2889.61). The parameters for [N_1111_][Arg] (*a*_2_, *b*_2_, *c*_2_ and *d*_2_) can also be calculated from a [N_1111_][Arg]-water system. However, when the model parameters of both MDEA and [N_1111_][Arg] are given, there are no adjustable parameters in the Weiland equation and the deviation between experiments and calculations will be significant. Thus, in this work the model parameters of [N_1111_][Arg] were regressed by fitting to the experiments of MDEA-[N_1111_][Arg] aqueous solutions. The optimized values are *a*_2_ = 0.2391, *b*_2_ = 0.8127, *c*_2_ = −0.5879, *d*_2_ = −0.7844. The ARD is 2.23%.

**Figure 4:**
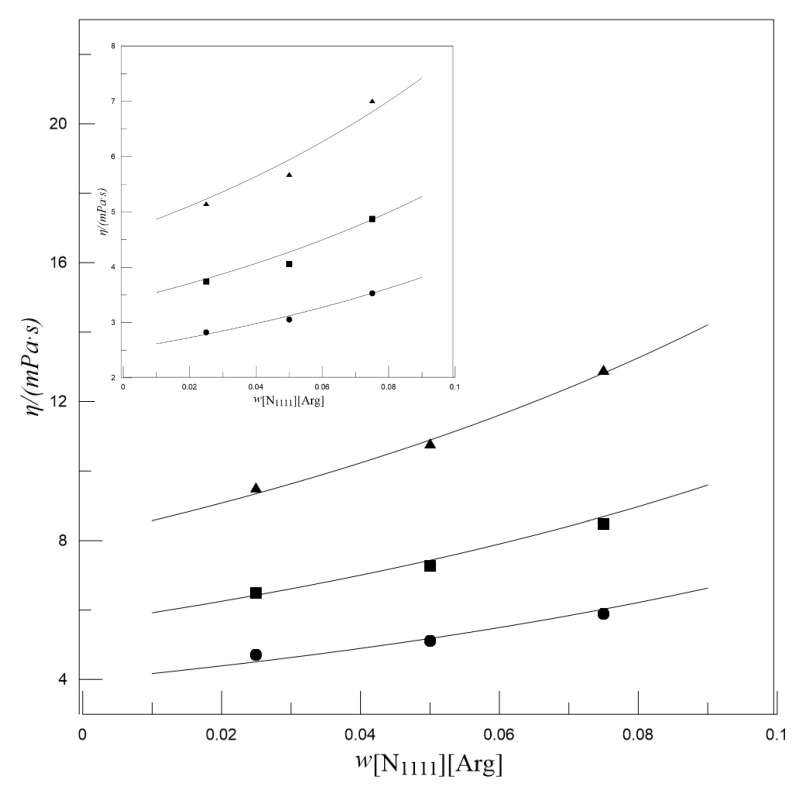
Effect of *w*_[N1111][Arg]_ on the viscosity of MDEA-[N_1111_][Arg] aqueous solutions at *w*_MDEA_ = 0.500, *w*_MDEA_ = 0.400 (inset), ▲*T* = 303.2 K; ■*T* = 313.2 K; ●*T* = 323.2 K. Symbols: Experimental values. Lines: Calculated values.

**Figure 5:**
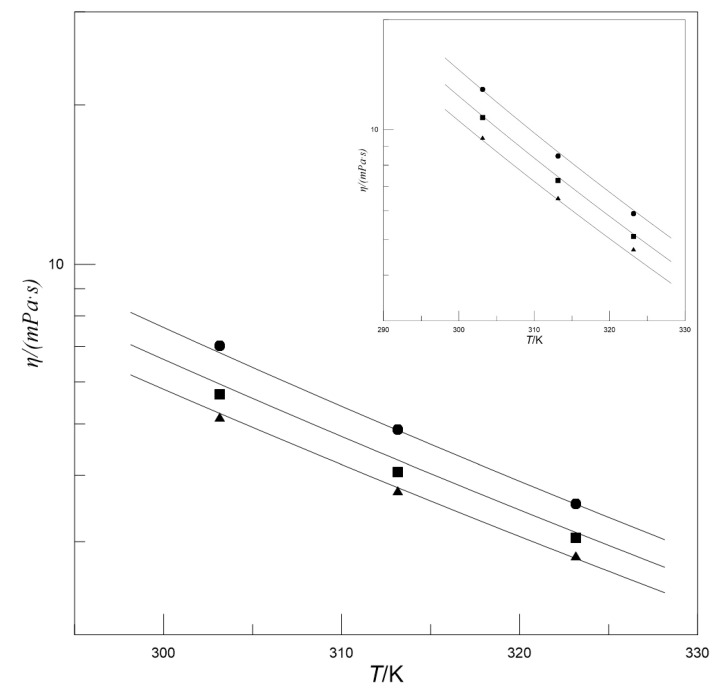
Effect of temperature on the viscosity of MDEA-[N_1111_][Arg] aqueous solutions at *w*_MDEA_ = 0.40, *w*_MDEA_ = 0.50 (insert), ▲*w*_[N1111][Arg]_ = 0.025; ■*w*_[N1111][Arg]_ = 0.050; ●*w*_[N1111][Arg]_ = 0.075. Symbols: Experimental values. Lines: Calculated values.

Figure 4 presents the influence of *w*_[N__1111][Arg]_ on the viscosity of MDEA-[N_1111_][Arg] aqueous solutions. It can be found that at a given *w*_MDEA_ and temperature, with the increase in *w*_[N__1111][Arg]_, the viscosity gradually increases and the amplitude of this increase gradually becomes larger. This may be caused by the larger molecular structure of [N_1111_][Arg], which affects the flow of the surrounding liquid. Therefore, although the *w*_[N__1111][Arg]_ is low, it has a significant impact on viscosity. Figure 5 presents the influence of temperature on the viscosity of MDEA-[N_1111_][Arg] aqueous solutions, and shows that the viscosity decreases with increasing temperature at given *w*_MDEA_ and *w*_[N__1111][Arg]_. This phenomenon may be explained by the expansion of the liquid with increasing temperature, which causes an increase in the molecular distance and a decrease in viscosity.

The viscosity activation energies (*Ea*) indicate the difficulty of material flow and can also reflect the sensitivity of viscosity to temperature changes. In this work, it was calculated by fitting the viscosity data using the following equation [46,47]:

(13) $$\eta =\eta_{\infty }exp\left(-Ea/RT\right)$$

where $\eta_{\infty }$ is the viscosity at infinite temperature, *R* is the gas constant. Equation (14) was used to linearly fit the viscosity data shown in Table 3. Then the *Ea* values can be obtained by the slope of the fitted line:

(14) $$\ln\left(\eta \right)=\frac{-Ea}{RT}+\ln\left(\eta_{\infty }\right)$$

The *Ea* value shown in Table 4 increased from 20.6 to 32.0 kJ·mol^−1^ with the increasing *w*_MDEA_ and w_[N1111][Arg]_. This implies that the higher the viscosity of the solution, the higher the viscosity activation energy. Although higher w_[N1111][Arg]_ can improve the absorption capacity of H_2_S, it also weakens the mass transfer. The calculated *Ea* value in this study is larger than that of water (*Ea*_water_ = 17.0 kJ·mol^−1^), but smaller than those of some common imidazolium-based ILs (e.g., *Ea*_[bmim][PF6]_ = 34.1 kJ·mol^−1^) [48].

In the process of absorbing H_2_S, the diffusion coefficient is also a significant parameter, and is highly important for the study of the gas–liquid mass transfer process. The Stokes–Einstein equation can express the relationship between diffusion coefficient and temperature and viscosity. It is generally accepted that the diffusion coefficient of a gas is inversely proportional to the viscosity of the solution [49].

Geert et al. [50] proposed a modified Stokes–Einstein relationship when studying the diffusion coefficient of N_2_O:

(15) $${\left(D_{N_{2}O}\eta^{0.8}\right)}_{solu}=\mathrm{constant}={\left(D_{N_{2}O}\eta^{0.8}\right)}_{water}$$

Portugal et al. [49] proposed that the ratio of the diffusivity of a gas in an electrolyte solution to the diffusivity of the same gas in water does not vary significantly with the nature of the diffusant. Therefore, it is reasonable to use the so-called N_2_O analogy to estimate the diffusion coefficient of H_2_S in solutions:

(16) $$\frac{D_{N_{2}O,\;\;solu}}{D_{N_{2}O,w}}=\frac{D_{H_{2}S,\;solu}}{D_{H_{2}S,w}}$$

Combined with the above two equations, it can be obtained that [49,51]:

(17) $$D_{H_{2}S,\;\;solu}=D_{H_{2}S,w}{\left(\frac{\eta_{water}}{\eta_{solu}}\right)}^{0.8}$$

where *η_solu_* is the viscosity of MDEA-[N_1111_][Arg] solution. *D_H_*_2*S,w*_ is the diffusivity of H_2_S in water. It can be fitted as a function of temperature according to the method of Versteeg et al. [51] using data from published studies by Haimour et al. [52] and Tamimi et al. [53]:

(18) $$D_{w}=6.04\times 10^{-7}\exp\left(-1714/T\right)\;m^{2}\cdot s^{-1}$$

The results are shown in Table 5. It can be seen that *D*_H2S_ decreases with the increase in *w*_MDEA_ and w_[N1111][Arg]_ at a given temperature, and at a given mass fraction, it increases with the increase in temperature. This indicates that lower mass fraction and higher temperature are favorable for the diffusion of H_2_S in MDEA-[N_1111_][Arg] solution.

## 4. Conclusions

In the present study, the viscosity and surface tension values of MDEA-[N_1111_][Arg] aqueous solutions were measured, and thermodynamic models were used to fit the experimental data. The experimental results and models were used to explore the effects of the solution mass fraction and temperature on the viscosity and surface tension. Furthermore, the *S^S^* and *H^S^* values of the solutions were obtained using the fitted model of the surface tension. The viscosity activation energy and the diffusion coefficient of H_2_S were calculated based on the measurement of viscosity. The main findings were as follows:

1. The surface tension decreased with the increase in solution mass fraction and temperature. The viscosity increased with the increase in solution mass fraction and decreased with the increase in temperature.
2. The thermodynamic models accurately reflected the effects of the solution mass fraction and temperature on the surface tension and viscosity.
3. With the increase in *w*_MDEA_, both the *S^S^* and *H^S^* decreased, whereas the *S^S^* increased and the *H^S^* decreased with the increase in *w*_[N1111][Arg]_.
4. The increase in solution mass fraction can result in the increase in *Ea* and decrease in *D*_H2S__,solu_.

## Figures and Tables

**Table 1:** Sample description.

Chemical Name	CAS	Purity (Mass Fraction, as Stated by the Supplier)	Source
MDEA	105-59-9	≥0.98	Aladdin Reagent, Shanghai, China
[N_1111_][Arg]	1450589-45-3	≥0.98	Shanghai Cheng Jie Chemical Co., Ltd., Shanghai, China
water	7732-18-5	Electrical resistivity > 15 MΩ cm at *T* = 298 K	Heal Force ROE-100 apparatus, Shanghai, China

**Table 2:** Surface tensions (*γ*) of N-methyldiethanolamine (MDEA)-[N_1111_][Arg] aqueous solutions under different mass fractions of MDEA (*w*_MDEA_), [N_1111_][Arg] (*w*_[N1111][Arg]_), and temperature ^a^.

*w*_MDEA_	*w*_[N1111][Arg]_	*γ/*(mN·m^−1^)		
		*T* = 303.2 K	*T* = 313.2 K	*T* = 323.2 K
0.300	0.025	52.2	50.0	47.8
	0.050	51.8	49.5	47.5
	0.075	51.5	49.2	47.2
0.400	0.025	50.1	48.2	46.1
	0.050	49.5	47.6	45.8
	0.075	49.0	47.2	45.5
0.500	0.025	47.4	45.9	43.8
	0.050	46.9	45.6	43.6
	0.075	46.3	45.4	43.3

^a^ standard uncertainties *u* are *u*(*T*) = 0.1 K; *u*(*w*_MDEA_) = 0.011; *u*(*w*_[N1111][Arg]_) = 0.002; *u*(*γ*) = 0.2 mN·m^−1^.

**Table 3:** Viscosities (*η*) of MDEA-[N_1111_][Arg] aqueous solutions under different mass fractions of MDEA (*w*_MDEA_), [N_1111_][Arg] (*w*_[N1111][Arg]_) and temperature ^a^.

*w*_MDEA_	*w*_[N1111][Arg]_	*η/*(mPa·s)		
		*T* = 303.2 K	*T* = 313.2 K	*T* = 323.2 K
0.300	0.025	3.10	2.35	1.87
	0.050	3.49	2.61	2.02
	0.075	4.03	2.96	2.26
0.400	0.025	5.15	3.74	2.82
	0.050	5.68	4.06	3.05
	0.075	7.01	4.87	3.53
0.500	0.025	9.52	6.50	4.71
	0.050	10.80	7.26	5.10
	0.075	12.90	8.47	5.89

^a^ standard uncertainties *u* are *u*(*T*) = 0.1 K; *u*(*w*_MDEA_) = 0.011; *u*(*w*_[N1111][Arg]_) = 0.002; and relative uncertainty *u_r_*(*η*) = 2%.

**Table 4:** Fitted Arrhenius parameters of MDEA-[N_1111_][Arg] aqueous solution under different mass fractions of MDEA (*w*_MDEA_) and [N_1111_][Arg] (*w*_[N1111][Arg]_) ^a^.

*w*_MDEA_	*w*_[N1111][Arg]_	*E*a/(kJ·mol^−1^)
0.300	0.025	20.6
	0.050	22.3
	0.075	23.6
0.400	0.025	24.5
	0.050	25.3
	0.075	28.0
0.500	0.025	28.7
	0.050	30.6
	0.075	32.0

^a^ Standard uncertainties *u* are *u*(*w*_MDEA_) = 0.011; *u*(*w*_[N1111][Arg]_) = 0.002; and relative uncertainty *u_r_*(*E*a) = 2%.

**Table 5:** Diffusion coefficient of H_2_S in MDEA-[N_1111_][Arg] solutions under different mass fraction of MDEA (*w*_MDEA_) and [N_1111_][Arg] (*w*_[N1111][Arg]_) ^a^.

*w*_MDEA_	*w*_[N1111][Arg]_	$D_{H_{2}S}$ $/(\times 10^{-9}m^{2}\cdot s^{-1})$		
		*T* = 303.2 K	*T* = 313.2 K	*T* = 323.2 K
0.300	0.025	0.72	0.91	1.13
	0.050	0.65	0.84	1.06
	0.075	0.58	0.76	0.97
0.400	0.025	0.48	0.63	0.81
	0.050	0.44	0.59	0.76
	0.075	0.37	0.51	0.68
0.500	0.025	0.29	0.41	0.54
	0.050	0.26	0.37	0.51
	0.075	0.23	0.33	0.45

^a^ Standard uncertainties *u* are *u*(*w*_MDEA_) = 0.011; *u*(*w*_[N1111][Arg]_) = 0.002; and relative uncertainty *u_r_*(*D*_H2S_) = 2%.
